# Supplementary material for: Development of a 3D atlas of the embryonic pancreas for topological and quantitative analysis of heterologous cell interactions
Source: Development. 2022 Feb 4;149(3):dev199655. doi: 10.1242/dev.199655 (PMC8918780; doi:10.1242/dev.199655)
Supplement: Supplementary information [file develop-149-199655-s1.pdf]

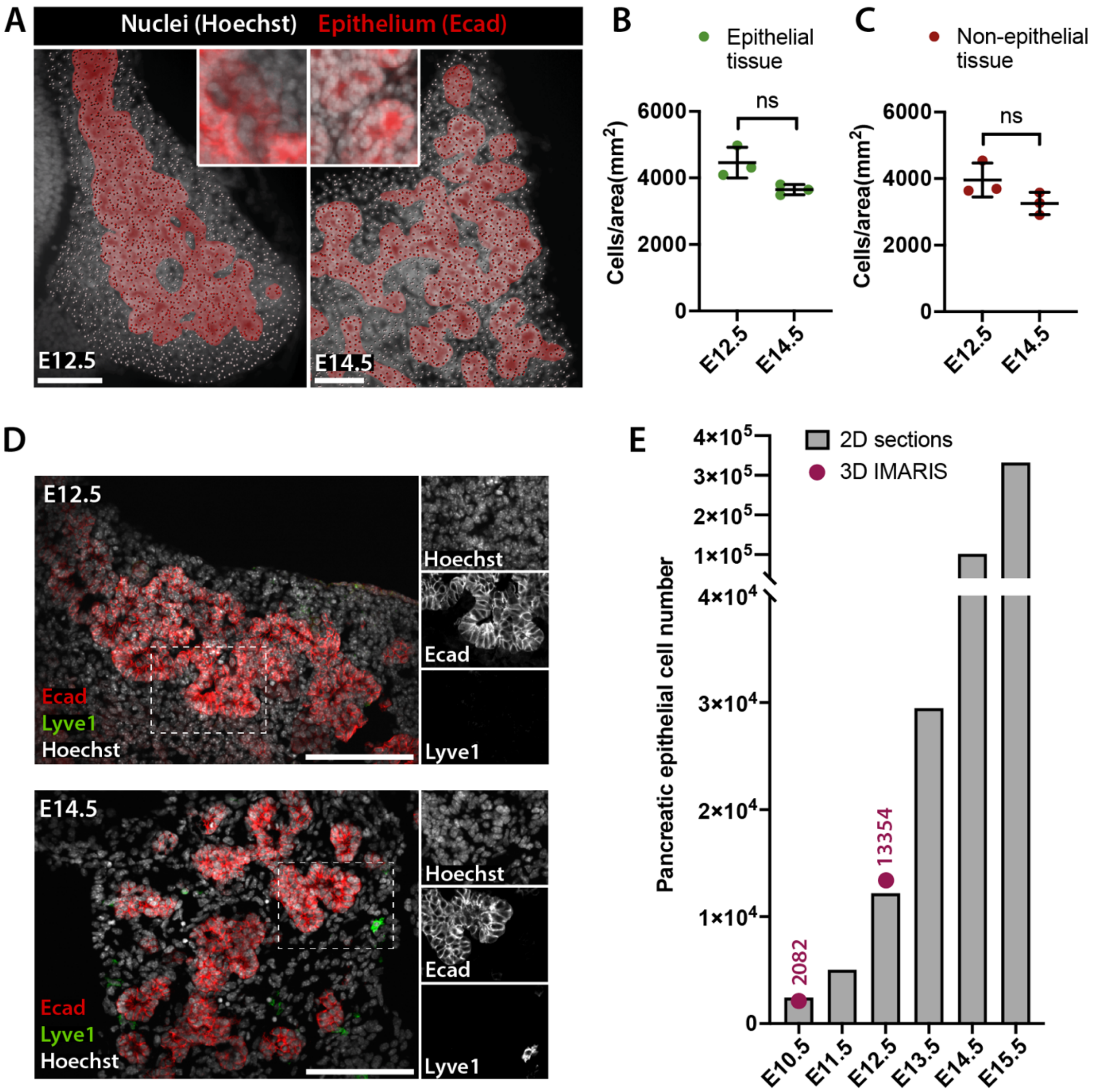

Fig. S1. Analysis of LSFM scans of embryonic pancreas.

(A) Cell density in pancreatic epithelium (Ecad^{+}) and non-epithelial tissues was measured on optical sections from LSFM scans of E12.5 and E14.5 embryonic pancreata using the Imaris software. Tissues were stained with antibodies against Ecad to distinguish the pancreatic epithelium and Hoechst as nuclear counterstain. Scale bar, 50 µm. **(B, C)** Scatter plot showing quantification of cells per area in epithelial (B) and non-epithelial tissues (C) at E12.5 (n=3) and E14.5 (n=3). Epithelial and non-epithelial areas were measured using the manual 'Surface creation' module and nuclei were detected using the 'Spots creation' module. For each embryo,

Development • Supplementary information

optical sections were analysed at regular intervals (E12.5, every 30µm: E14.5, every 60µm) throughout the entire pancreatic tissue to calculate the average cell density. Overall cell density in epithelial and non-epithelial pancreatic tissues was unchanged between E12.5 and E14.5 pancreata. **(D)** Representative IF images of pancreatic tissue sections at E12.5 (A) and E14.5 (B). IF labelling for the Lymphatic vessel endothelial hyaluronan receptor 1 (Lyve1; green) marks rare lymphatic cells in the embryonic pancreas. Ecad (red) demarcates the pancreatic epithelium. Hoechst (grey) was used as nuclear counterstain. Insets show higher magnifications of the boxed regions as single channel configuration. Scale bars, 100 µm. **(E)** Measurement of epithelial cell number in the dorsal pancreas from E10.5 to E15.5 using Halo software and Imaris software. Total epithelial cell number was determined with HALO software by integrating the number of epithelial cells ($Ecad^{+}$) computed on regularly spaced 2D sections (every 30 µm) spanning the whole pancreatic anlages (Gonay et al., 2021). 3D Imaris quantification (red) was performed using 'Spots' segmentation on epithelial cells ($Ecad^{+}$ or $Pdx1^{+}$) of the entire pancreatic tissue at stage E10.5 and E12.5.

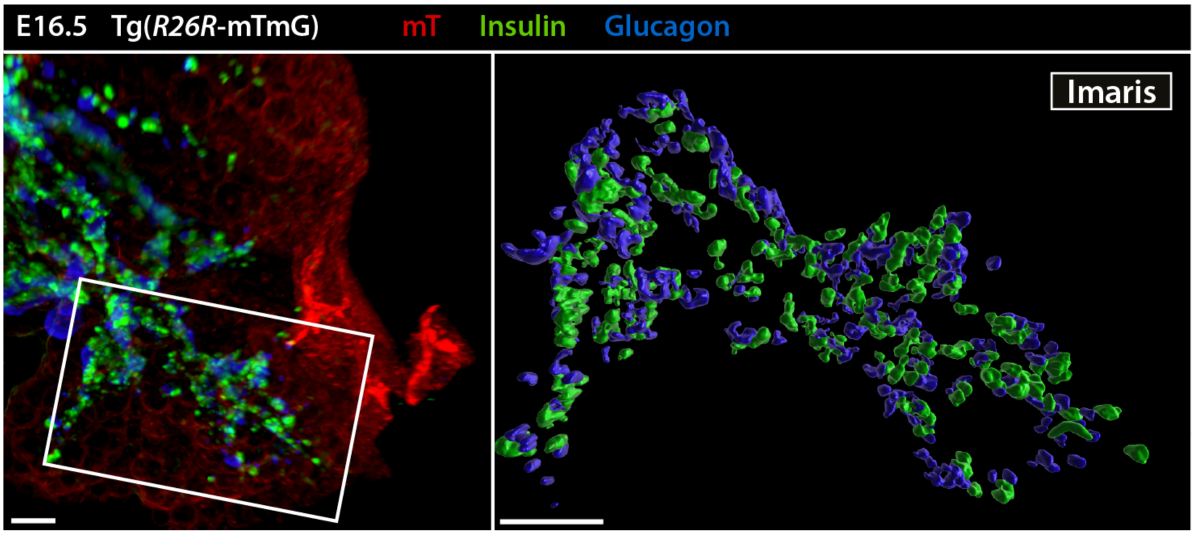

Fig. S2. LSFM images of pancreas endocrine tissue. Representative LSFM 3D image (left) and Imaris surface rendering (right) of the pancreas from Tg(*R26R*-mTmG) embryos at E16.5. WMIF for insulin (green) and glucagon (blue) labels the pancreatic beta- and alpha- endocrine cells, respectively. mT (red) mark all cell membranes. Right panel, surface rendered 3D model of the boxed area showing insulin$^+$ and glucagon$^+$ cells adjacent to each other, arranged in a peninsular structure with peripheral glucagon$^+$ cells and internal insulin$^+$ cells. Scale bars, 100 µm.

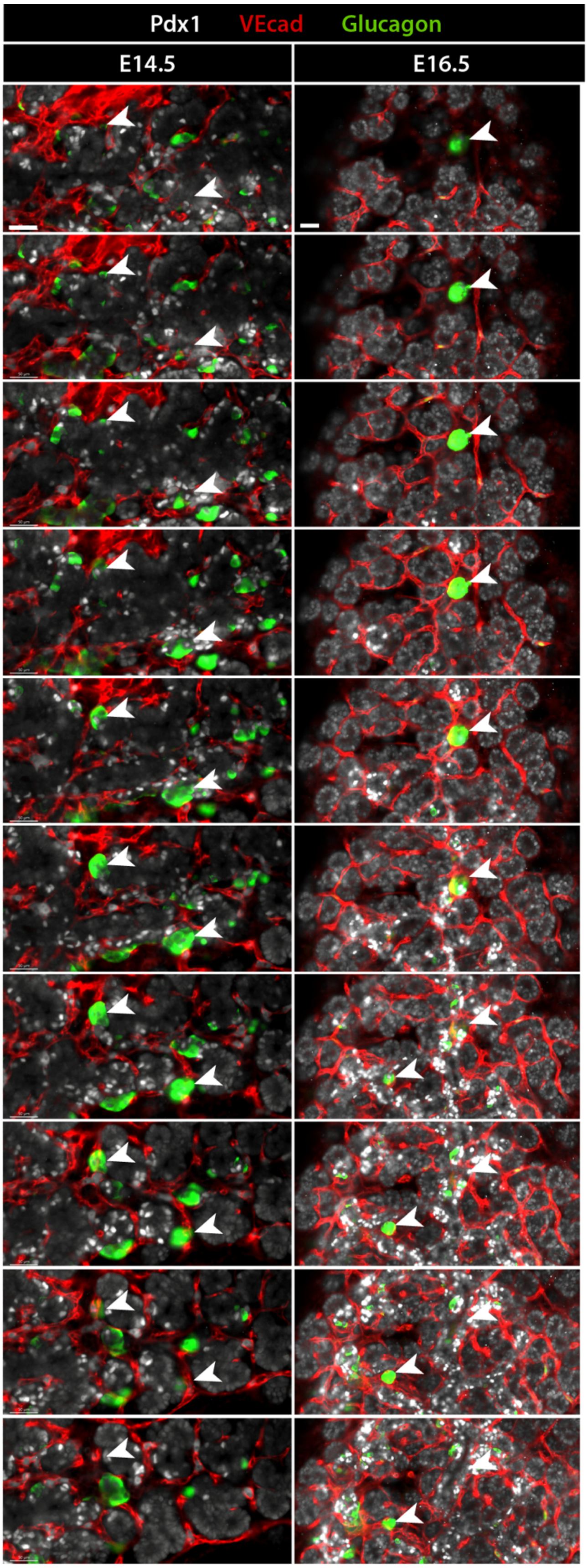

Fig. S3. LSFM scans of pancreas endocrine tissue. Representative consecutive optical sections (separated by 10 µm) through E14.5 and E16.5 pancreas after LSFM imaging, showing Pdx1^{+} pancreatic cells (grey), VEcad^{+} endothelial cells (red) and glucagon+ cells (green). Arrowheads indicate glucagon^{+} clusters arising from the epithelium surrounded by vessels. Scale bar, 50 µm.

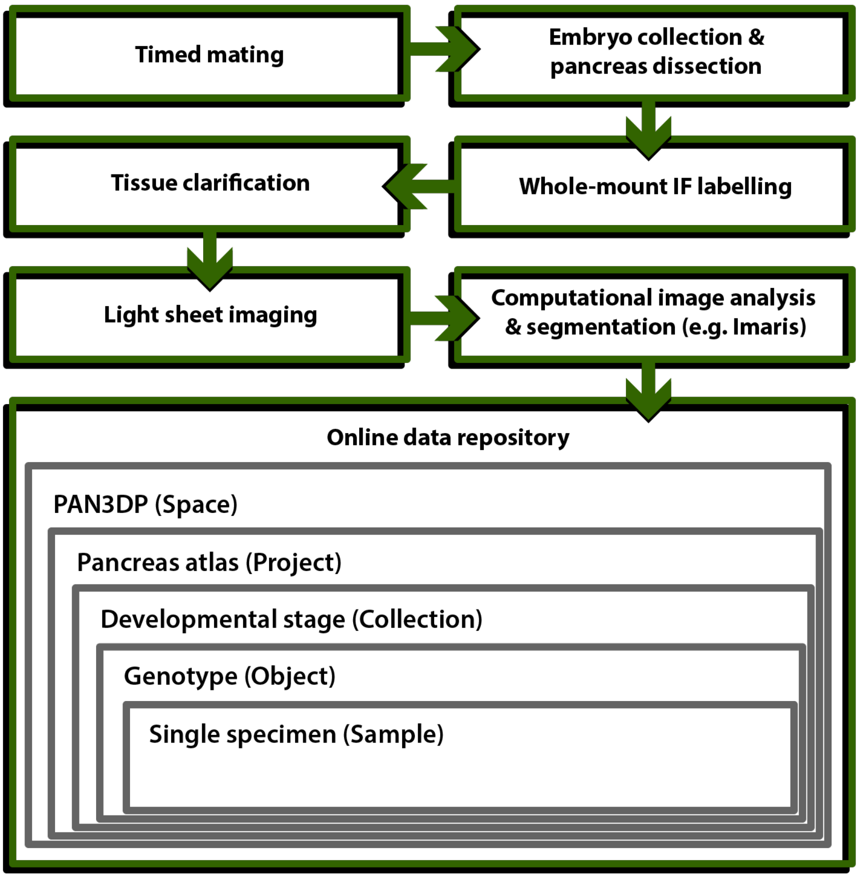

Fig. S4. Schematic representation of data collection and deposition. Flowchart describing the experimental steps used to generate the image dataset presented here and the organizational structure of the online data repository in which they can be accessed.

Development • Supplementary information

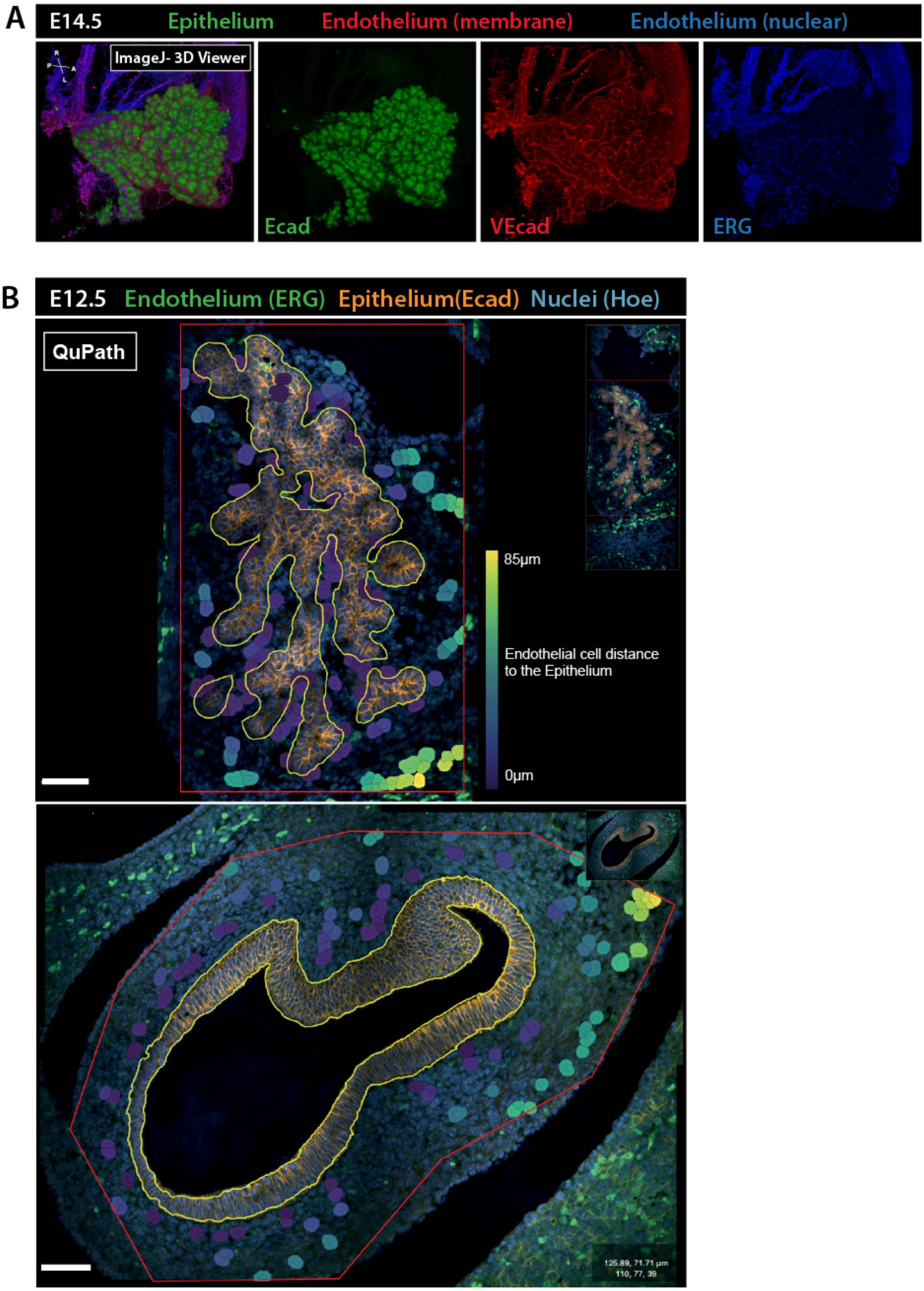

Development • Supplementary information

Fig. S5. Visualisation and analysis of LSFM 3D images of pancreatic tissue using open- source software. (A) Representative LSFM 3D images of the E14.5 wildtype pancreas shown in Fig. 3B visualized using the 3D Viewer plugin in ImageJ. WMIF for Ecad (green), VEcad (red) and ERG (blue). 3D images and surface renderings are shown as merged (leftmost panel) and individual channels (right panels). A, anterior; P, posterior; L, left; R, right. **(B)** Quantitative analysis of endothelial cell distribution around the pancreatic epithelium using QuPath. QuPath rendering of the E12.5 pancreas and stomach sections shown in Fig. 4G. E- cadherin (Ecad) labelling is shown in orange, ERG in green and Hoechst in blue. ERG positive endothelial cells are pseudocolored using the "Distance to annotation" tool. Endothelial cells pseudocolour follow the Viridis palette according to their distance from the epithelium ($Ecad^{+}$ region).

Table S1. Data deposited in the Pancreas Embryonic Cell Atlas.

Stage	All nuclei		Epithelial cells			Endothelial cells				Mesenchymal cells		Endocrine Cells				No of images
	DRAQ5	HOECHST	Pdx1	Pdx1-Cre mTmG	E-Cadherin	VE-Cadherin	ERG	Sox17	Cdh5-Cre mTmG	Nkx2.5-Cre mTmG	Nkx2.5-Cre H2B	Glucagon	Ins2-Cre mTmG	Insulin	Pax6	
E12.5					488 nm	568 nm										3
E12.5		405 nm	568 nm		488 nm											7
E12.5			568 nm			488 nm										1
E12.5			647 nm	488 nm												4
E12.5			488 nm					568 nm								1
E12.5					647 nm			568 nm								3
E12.5			647 nm							488 nm						6
E12.5		405 nm			488 nm	568 nm	647 nm									2
E12.5		405 nm	568 nm			488 nm										1
E12.5		405 nm			647 nm								488 nm			4
E12.5		405 nm			647 nm				488 nm							3
E14.5		405 nm			488 nm	568 nm	647 nm									4
E14.5	647 nm		568 nm			488 nm										4
E14.5			647 nm							488 nm						5
E14.5					488 nm	568 nm	647 nm									3
E14.5		405 nm	488 nm			568 nm						647 nm				2
E14.5		405 nm	647 nm			568 nm									488 nm	1
E14.5		405 nm				568 nm					488 nm	647 nm				3
E16.5		405 nm	647 nm			568 nm						488 nm				2
E16.5		405 nm				568 nm					488 nm	647 nm				2
E16.5		405 nm										647 nm		488 nm		1
E16.5		405 nm	647 nm			568 nm									488 nm	1
E16.5		405 nm										647 nm	488 nm			1
E18.5		405 nm	488 nm			568 nm						647 nm				1
P0		405 nm	647 nm			568 nm						488 nm				1

Colour	Wavelength
(blue)	405 nm
(green)	488 nm
(orange)	568 nm
(purple)	647 nm

Table S2. Antibodies and fluorescent dyes

Name	Company	Catalog Number	Dilution
Rat anti-E-CADHERIN	Sigma	U3254	1:500
Rabbit anti-ERG	Abcam	Ab92513	1:100
Chicken anti-GFP	Aves	GFP-1020	1:400
Rabbit anti-Glucagon	Immunostar Inc.	20076	1:500
Guinea pig anti-Insulin	Agilent/Dako	A0564	1:300
Guinea pig anti-PDX1	Abcam	Ab47308	1:500
Rat anti-RFP	Antikörper online	ABIN334653	1:400
Goat anti-SOX17	R&D Systems	AF1924	1:200
Goat anti-VE-CADHERIN	R&D Systems	AF1002-SP	1:50
Donkey Alexa Fluor 488-labelled Anti-Goat IgG	Invitrogen	A11055	1:1000
Donkey Alexa Fluor 488-labelled Anti-Guinea Pig IgG	Dianova	706-545-148	1:1000
Donkey Alexa Fluor 488-labelled Anti-Rat IgG	Invitrogen	A21208	1:1000
Goat Alexa Fluor 488-labelled Anti-Chicken IgG	Invitrogen	A11039	1:1000
Donkey Alexa Fluor 568-labelled Anti-Goat IgG	Invitrogen	A11057	1:1000
Donkey Alexa Fluor 594-labelled Anti-Rat IgG	Invitrogen	A21209	1:1000
Goat Alexa Fluor 568-labelled Anti-Guinea Pig IgG	Invitrogen	A11075	1:1000
Donkey Alexa Fluor 647-labelled Anti-Guinea Pig IgG	Dianova	706-605-148	1:1000
Donkey Alexa Fluor 647-labelled Anti-Rabbit IgG	Invitrogen	A31573	1:1000
Donkey Alexa Fluor 647-labelled Anti-Rat IgG	Dianova	712-605-153	1:1000
eBioscience™ DRAQ5™	Invitrogen	65-0880-96	1:500
Hoechst 33342	Invitrogen	H1399	1:1000

Development • Supplementary information
