# Supplementary material for: Development of a 3D atlas of the embryonic pancreas for topological and quantitative analysis of heterologous cell interactions
Source: Development. 2022 Feb 4;149(3):dev199655. doi: 10.1242/dev.199655 (PMC8918780; doi:10.1242/dev.199655)
Supplement: Reviewer comments [file develop-149-199655_review_history.pdf]

Development of a 3D atlas of the embryonic pancreas for topological and quantitative analysis of heterologous cell interactions

Laura Glorieux, Aleksandra Sapala, David Willnow, Manon Moulis, Anna Salowka, Jean-Francois Darrigrand, Shlomit Edri, Anat Schonblum, Lina Sakhneny, Laura Schaumann, Harold F. Gómez, Christine Lang, Lisa Conrad, Fabien Guillemot, Shulamit Levenberg, Limor Landsman, Dagmar Iber, Christophe E. Pierreux and Francesca M. Spagnoli
DOI: 10.1242/dev.199655

Editor: James Wells

Review timeline

Original submission:	30 March 2021
Editorial decision:	8 June 2021
Rebuttal received:	13 June 2021
Editorial decision:	14 June 2021
First revision received:	26 November 2021
Accepted:	20 December 2021

Original submission

First decision letter

MS ID#: DEVELOP/2021/199655

MS TITLE: Development of a 3D atlas of the embryonic pancreas for topological and quantitative analysis of heterologous cell interactions

AUTHORS: Laura Glorieux, Aleksandra Sapala, David Willnow, Manon Moulis, Shlomit Edri, Jean-Francois Darrigrand, Anat Schonblum, Lina Sakhneny, Laura Schaumann, Harold F Gomez, Christine Lang, Lisa Conrad, Fabien Guillemot, Shulamit Levenberg, Limor Landsman, Dagmar Iber, Christophe E Pierreux, and Francesca M Spagnoli

Dear Dr. Spagnoli,

I have now received all the referees' reports on the above manuscript, and have reached a decision. I am sorry to say that the outcome is not a positive one. The referees' comments are appended below, or you can access them online: please go to Development's submission site site and click on the 'Manuscripts with Decisions' queue in the Author Area.

As you will see, the referees raise some significant concerns about your paper, and are not strongly in favour of publication. Having looked at the manuscript myself, I agree with their views, and I must therefore, reject your paper. While the referees felt that the use of light sheet microscopy was novel, they still felt that even as a resource article the data were not enough of an advance over previous studies to warrant publication in Development.

I do realise this is disappointing news, but Development receives many more papers than we can publish, and we can only accept manuscripts that receive strong support from referees.

I do hope you find the comments of the referees helpful, and that this decision will not dissuade you from considering Development for publication of your future work. Many thanks for sending your manuscript to Development.

© 2022. Published by The Company of Biologists under the terms of the Creative Commons Attribution License (https://creativecommons.org/licenses/by/4.0/).

Reviewer 1

Advance summary and potential significance to field

In this article, several groups report the production of three-dimensional images of pancreas development by light-sheet microscopy, their analysis and their availability in a repository.
The article is well written and the images and analysis are of high quality.
However, the study is more descriptive than the articles usually published in Development and of limited scope (two developmental stages only). The technical novelty resides in the first use of CUBIC for tissue clearing of the pancreas but it has been used for many other tissues. The use of light sheet microscopy to image pancreatic samples is novel but at this stage of development and even later the pancreas can also be analysed by confocal or two-photon microscopy (though the imaging times are longer). 3D imaging of the pancreas has been used in numerous studies, some of which are reported in the discussion. These studies used different 3D staining and imaging modalities but also produced data of quality. The analyses are novel (distance of cells to epithelium) but mostly use a software already largely used by the community (Imaris). Biological novelty is also limited. Its merit is the analysis of the percentage of endothelial epithelial and mesenchymal cells, extending previous work from L. Landsman focusing on epithelial and mesenchymal cells. It also reports that endothelial and mesenchyme cells get closer to the epithelium between E12.5 and E14.5. This is of potential interest, but would merit mechanistic investigations.
An excellent initiative is to make the data available to the scientific community.

Comments for the author

With regards to the observation that endothelial and mesenchymal cells get closer to the epithelium between E12.5 and E14.5, it would be interesting to know whether there is increased tissue packing between these two stages, notably in the mesenchyme.

Reviewer 2

Advance summary and potential significance to field

This paper (intended to be a Techniques and Resources Article) reports a description of mouse pancreas development at embryonic days 12.5 and 14.5, using light sheet microscopy to generate a 3-dimensional view. The authors made extensive use of fluorescent reporter transgenes driven by tissue-specific cre recombinase, taking advantage of the mTmG Cre reporter system, which labels cell boundaries and can identify cell-cell contacts. The accuracy of the fluorescent reporter system was validated by comparisons to immunostaining. Fluorescent reporters are sometimes combined with immunostaining for endogenous proteins.

The key concrete novel finding is technical - the convincing demonstration that light sheet fluorescent microscopy allows to generate confocal-grade images of the growing pancreas, while preserving 3-dimentional structure. This will be a useful technique for groups studying organ formation, in the pancreas and other organs.
Beyond this, it presents descriptive data. This is a good thing, as it provides the community with a solid foundation to study aspects of pancreas development that are still incompletely understood, and the authors are to be commended for their embryonic cell atlas resource.

Comments for the author

I would encourage the authors to report something new about our understanding of pancreas development, which has emerged from their analysis. There have been many comprehensive reviews of pancreas development, many of which are not cited, for example pmid 17881611. What are the novel findings here?
The authors mention here and there some interesting observations e.g. different blood vessel density between the embryonic pancreas and the stomach (which cannot be “functional” as both organs are not functioning at this stage; perhaps related to the more complex folding and multi-layering of epithelium in the pancreas?),

© 2022. Published by The Company of Biologists under the terms of the Creative Commons Attribution License (https://creativecommons.org/licenses/by/4.0/).

increased blood vessel density with age, and an interesting stability of relative epithelial / mesenchymal / endothelial cell ratios over time. However this feels too sketchy.

Specific comments and suggestions
1. The authors could potentially use their tools and perhaps even existing data to extract high resolution quantitative information about 3D structure and cell numbers in developing islets, ducts and acini. This is important, and is difficult to obtain using previous methods.
2. It can be interesting and feasible to use the same technology for imaging the structure of the mature (adult or even newborn) pancreas, and contrasting it with earlier stages (e.g. abundance and organization of mesenchyme and blood vessels vs the epithelium; cell size; cell shape).
3. An assessment of total cell numbers in the pancreas at each stage is also important yet missing from literature, and can perhaps be inferred from the data.
4. How can vascular density increase (figure 4) while the proportion of endothelial cells remains the same (7%, line 267)? Can this is the result of increased epithelial cell size? More ECM?
5. Introduction – I suggest to elaborate a bit more about tubulogenesis and lumen formation (e.g. 19914171). This is a key step preceding the tip-trunk stage.
6. The final paragraph about bioprinting is out of context. It is not clear at all why and how the data in this study will help bioprinting for cell therapy or disease modeling. Is there a reason to think that the exact structures identified here within fetal islets are applicable? If so, the authors should try and print such a structure from live sorted cells.

Reviewer 3

Advance summary and potential significance to field

Glorieux et al. report on the establishment of 3D image acquisition and analyses of E12.5 and E14.5 whole mount mouse pancreatic tissues. More specifically, they study cellular interactions between pancreatic epithelium (as labelled with Pdx1) on the one hand and endothelium (as labelled with Erg or Cdh5) and mesenchyme (as labelled with Nkx2.5) on the other hand. They found that from E12.5 to E14.5 the interaction between endothelium and epithelium gets more intimate (moving from a 5 to a 2.5 µm distance). The authors also observe that this interaction is stronger in the pancreas (60-80%) than stomach (10-20%). In addition mesenchymal cells also move a bit closer to the pancreatic epithelium (moving from a 7.5 to a 4.5 µm distance). The ratios of cell populations in 15 µm proximity to each other remain almost constant, but a trend towards an increase in endothelial cells and epithelial cells versus a trend towards a decrease in mesenchymal cells could be observed. The authors further observe that blood vessels close to the pancreatic epithelium increase in diameter, indicative of blood vascular perfusion. In sum, Glorieux et al. report on a technical advance in pancreatic imaging by using SPIM / LSFM that, however, has been applied to several other embryonic tissues (sometimes even using live cell imaging) – therefore, it is not well suited as a pure resources paper. The findings reported are of descriptive nature and thus limit the enthusiasm to publish this report in Development, in particular, since no major insights into the cellular and molecular mechanisms of pancreatic development are reported. However, the technology could nicely be combined with manipulative experiments (not shown by the authors though) as to give the manuscript more value in terms of using the technology to better understand mechanisms in development.

Comments for the author

Major issues:
(1) The authors only report on pancreatic epithelial – mesenchymal/endothelial cell interactions. They should extend their study on beta cell – mesenchymal/endothelial cell interactions using the Ins2-Cre line that they used only once in their study. Does this interaction change between E12.5 and E14.5?
(2) The authors must extend their study to blood vessel perfusion. Using dextran or tomato lectin (or another lectin) they could directly show whether the blood vessels are perfused at E14.5 (as they suggest) and, more importantly, whether beta cell development or endocrine progenitor cell development happens close to perfused rather than non-perfused blood vessels. Ngn3-Cre mice along with Ins2-Cre mice need to be used.

© 2022. Published by The Company of Biologists under the terms of the Creative Commons Attribution License (https://creativecommons.org/licenses/by/4.0/).

(3) Figure 3E and 4 are probably the Figures with the newest data. Is it possible to manipulate these aspects, e.g. block blood perfusion in the developing pancreas and see what happens?
(4) The authors must state which novel conclusions they actually draw from their 3D images; such conclusions must be mentioned in the abstract.

Minor issues:
(1) Figure 2C shows Ins2-Cre mice at E12.5, but no data on these mice at E14.5 are provided.
(2) Is it possible to visualize the birth of beta cells using LSFM live cell imaging? This is probably not easy but could give novel dynamic information about beta cell development.
(3) Any idea why there is more epithelial-endothelial cell interaction in pancreas versus stomach? Is it because there are fewer endocrine cells in the stomach versus pancreas?

Author rebuttal letter

Dear Dr. Wells,

I thank you for the careful evaluation of our manuscript entitled "Development of a 3D atlas of the embryonic pancreas for topological and quantitative analysis of heterologous cell interactions" [DEVELOP/2021/199655].

I understand the concerns that you have on the submitted version of our study. However, after having read the referees' comments with attention, I have decided to write to you an appeal for reconsideration of our manuscript. We feel that the overall evaluation of our manuscript is positive and the feedback very encouraging. It is true that our study is mostly descriptive, however the LSFM images online repository of the embryonic pancreas that we built is unique, not available for any other tissue. Indeed, all referees agree that "the embryonic cell atlas resource represents an excellent initiative" and find that our large dataset of LSFM images "provide the community with a solid foundation to study aspects of pancreas development".
Moreover, the referees' comments are very constructive, the experiments requested are appropriate and can be addressed by us within a short span of time. Our manuscript in its present form already includes some quantitative biological measurements, such the quantification of relative cellular abundance and distribution of distinct cellular components within the pancreatic tissue, at two embryonic stages. As suggested by the three referees, we will further expand these measurements at later time points and concentrate on the endocrine islets using the Ins2-Cre line, which is available to us. Also, some of the experiments suggested have been already performed by us, such as the assessment of the total cell numbers in the pancreas (see referee #2) or some of the manipulation of the vessels/blood perfusion (see referee #3). These results will be included in the revised manuscript.
I really hope the information provided above provides you with sufficient information and that you will give us the opportunity to revise and resubmit our manuscript to *Development*.

I would like to take this opportunity to thank you again for your valuable support and time. Looking forward to hearing from you soon.

Yours Sincerely,
Francesca

Francesca M. Spagnoli, MD PhD
Group Leader

© 2022. Published by The Company of Biologists under the terms of the Creative Commons Attribution License (https://creativecommons.org/licenses/by/4.0/).

Rebuttal response letter

MS ID#: DEVELOP/2021/199655

MS TITLE: Development of a 3D atlas of the embryonic pancreas for topological and quantitative analysis of heterologous cell interactions

AUTHORS: Laura Glorieux, Aleksandra Sapala, David Willnow, Manon Moulis, Shlomit Edri, Jean-Francois Darrigrand, Anat Schonblum, Lina Sakhneny, Laura Schaumann, Harold F Gomez, Christine Lang, Lisa Conrad, Fabien Guillemot, Shulamit Levenberg, Limor Landsman, Dagmar Iber, Christophe E PIERREUX, and Francesca M Spagnoli

Dear Dr. Spagnoli,

Thank you for your appeal on your recently rejected manuscript. I do understand your disappointment, but given the opinions expressed by the reviewers, I saw little option other than to decline the paper.

However, we are always willing to give authors the chance to defend their manuscripts, and I do recognise that you make some valid comments in your letter. Therefore we would be willing to reconsider a revised version of your manuscript that deals, as far as possible, with the points raised by the reviewers. Upon resubmission, please provide a detailed response to the reviewers' comments and highlighting particularly any concerns that have not been included in the revised manuscript.

The revised manuscript and rebuttal will be sent to the original reviewers (if they are still available). If they are convinced by your arguments, then we would be able to consider the manuscript for publication. In your rebuttal letter you outlined several new experiments that you intend to include in the revision, including later time points, additional data from the Ins-Cre lines and quantitative analysis of image data sets. In your revised manuscript and response to reviewers I encourage you to emphasize new findings in pancreatic development and how this work is an advance over other imaging studies of the pancreas.

First revision

Author response to reviewers' comments

Point-by-point response to reviewers’ concerns

We thank the reviewers for their insightful and helpful comments, as we feel addressing them has improved our manuscript significantly. Below is a point- by-point response to reviewers’ concerns and how we are in the process to address all of them, our responses are shown in blue.

Reviewer 1

In this article, several groups report the production of three-dimensional images of pancreas development by light-sheet microscopy, their analysis and their availability in a repository. The article is well written and the images and analysis are of high quality. However, the study is more descriptive than the articles usually published in Development and of limited scope (two developmental stages only).

The technical novelty resides in the first use of CUBIC for tissue clearing of the pancreas but it has been used for many other tissues. The use of light sheet microscopy to image pancreatic samples is novel but at this stage of development and even later the pancreas can also be analysed by confocal or two-photon microscopy (though the imaging times are longer).

3D imaging of the pancreas has been used in numerous studies, some of which are reported in the discussion. These studies used different 3D staining and imaging modalities but also produced data of quality.

The analyses are novel (distance of cells to epithelium) but mostly use a software already largely

© 2022. Published by The Company of Biologists under the terms of the Creative Commons Attribution License (https://creativecommons.org/licenses/by/4.0/).

used by the community (Imaris). Biological novelty is also limited. Its merit is the analysis of the percentage of endothelial, epithelial and mesenchymal cells, extending previous work from L. Landsman focusing on epithelial and mesenchymal cells. It also reports that endothelial and mesenchyme cells get closer to the epithelium between E12.5 and E14.5. This is of potential interest, but would merit mechanistic investigations. An excellent initiative is to make the data available to the scientific community.

We understand the concern of this reviewer about the limited scope and novelty of our study. It is true that our study is mostly descriptive, as this reviewer pointed out. However, the primary goal of this resource article is to present the pancreatic embryonic cell atlas resource, which has been praised for being an excellent initiative by all reviewers. The LSFM images online repository of the embryonic pancreas that we built is unique, not available for any other tissue.
In compliance with the reviewer's suggestions, we have expanded the scope of our study. In the revised manuscript, we have extended our analysis to additional time points (E16-5-p0) and concentrated on the developing endocrine islets and their interactions with the surrounding vessels and mesenchymal supporting lineages. The new experiments resulted into two new main figures (Figures 6 and 7) and 4 new Supplementary figures.

Comments for the Author:
With regards to the observation that endothelial and mesenchymal cells get closer to the epithelium between E12.5 and E14.5, it would be interesting to know whether there is increased tissue packing between these two stages, notably in the mesenchyme.

We thank the reviewer for raising this important point. To address whether cell compaction may be responsible for the increase in heterologous cell-cell interactions, we measured cell density in pancreatic epithelium and non- epithelial tissues using optical sections from LSFM scans.
We found that cell density in epithelial and non-epithelial pancreatic tissues is unchanged between E12.5 and E14.5. This new analysis has been added to new Fig. S1.

Reviewer 2
This paper (intended to be a Techniques and Resources Article) reports a description of mouse pancreas development at embryonic days 12.5 and 14.5, using light sheet microscopy to generate a 3-dimensional view. The authors made extensive use of fluorescent reporter transgenes driven by tissue-specific cre recombinase, taking advantage of the mTmG Cre reporter system, which labels cell boundaries and can identify cell-cell contacts. The accuracy of the fluorescent reporter system was validated by comparisons to immunostaining. Fluorescent reporters are sometimes combined with immunostaining for endogenous proteins.
The key concrete novel finding is technical - the convincing demonstration that light sheet fluorescent microscopy allows to generate confocal-grade images of the growing pancreas, while preserving 3-dimentional structure. This will be a useful technique for groups studying organ formation, in the pancreas and other organs. Beyond this, it presents descriptive data. This is a good thing, as it provides the community with a solid foundation to study aspects of pancreas development that are still incompletely understood, and the authors are to be commended for their embryonic cell atlas resource.

Comments for the Author:
I would encourage the authors to report something new about our understanding of pancreas development, which has emerged from their analysis. There have been many comprehensive reviews of pancreas development, many of which are not cited, for example pmid 17881611. What are the novel findings here?

We thank the reviewer for recognizing the value of our pancreatic embryonic cell atlas resource. We agree that other reviews describing embryonic pancreas in the mouse have been published. As suggested by the reviewer, we have now cited in our revised manuscript Jørgensen et al. 2007 (pmid: 17881611), which pioneered the field of 3D imaging of mouse embryonic pancreas using confocal microscopy, and a more recent review article from Alanentalo et al. 2021 (doi: 10.3389/fendo.2021.633063) on OPT and LSFM imaging of the pancreas. So far, most of the imaging studies have been carried out on adult pancreas instead of embryonic tissue. Moreover, differently from previous studies, our image acquisition and quantitative analyses focused on the

© 2022. Published by The Company of Biologists under the terms of the Creative Commons Attribution License (https://creativecommons.org/licenses/by/4.0/).

3D spatial organization of the pancreatic epithelium in a broader anatomical context and in relation with its associated tissues (mesenchyme and vessels). The LSFM technique has enabled us to assess various quantitative and spatial features of pancreatic organogenesis at a resolution that has never been done before, including cellular ratio and distribution of different populations as well as vessel diameter. These quantitative analyses have been further expanded in the revised manuscript (see also below Answers to point 1, 2 of this reviewer).

Specific comments and suggestions:

1. The authors could potentially use their tools and perhaps even existing data to extract high resolution quantitative information about 3D structure and cell numbers in developing islets, ducts and acini. This is important, and is difficult to obtain using previous methods.

In the revised manuscript, we expanded our analysis to additional time points (E16.5, p0) and concentrated on the endocrine islets structure and the surrounding vessels and mesenchymal supporting lineages. The new image datasets have been used to define the 3D structure of the developing endocrine islets and to measure their vascularization (size and density of surrounding vessels) and interaction with mesenchymal cells. The new data are shown in new Figures 6 and 7 and new Figures S2 and S3.

2. It can be interesting and feasible to use the same technology for imaging the structure of the mature (adult or even newborn) pancreas, and contrasting it with earlier stages (e.g. abundance and organization of mesenchyme and blood vessels vs the epithelium; cell size; cell shape).

In compliance with the reviewer's request, we applied the same LSFM technique to image pancreatic tissue from E16.5, E18.5 embryos and newborns. We showed that it is possible to adapt the whole-mount labelling and imaging to later stages (up to p0). Qualitative images of the newborn endocrine pancreas have been included in new Figure 6 and uploaded in the pancreatic embryonic cell atlas resource. Future work will expand the analysis to adult pancreatic tissue.

3. An assessment of total cell numbers in the pancreas at each stage is also important yet missing from literature, and can perhaps be inferred from the data.

We followed the suggestion of the reviewer and measured total cell numbers in the embryonic pancreas in the window of the time between E10.5 and E15.5 on sections using HALO software and on LSFM images using Imaris software. The new data have been included in new Figure S1.

4. How can vascular density increase (figure 4) while the proportion of endothelial cells remains the same (7%, line 267)? Can this is the result of increased epithelial cell size? More ECM?

We thank the reviewer for this comment. It is true that these two observations seem counterintuitive, but they can be explained by the following arguments. First, between E12.5 and E14.5 endothelial cells progressively get closer to the epithelial surface, resulting into an increase of the vascular density within the first few micrometers around the epithelial surface, but the overall vascular density does not change when one considers the entire 15 µm region surrounding the epithelial surface (see Figure 4 C and E). This has been now specified in the Result section page 10. Second, the proportion of epithelial, endothelial and mesenchymal cells remains stable, because the three populations expand proportionately between E12.5 and E14.5 within the analyzed region. Indeed, we found that from E12.5 to E14.5, the number of epithelial cells increases from ~12.000 to ~100.000 cells (see new Figure S1E). In the same window of time, we also measured that the absolute number of endothelial cells in the 15 µm region increases from ~800 at E12.5 to ~8.000 at E14.5 (data not shown). Extrapolation for the mesenchymal population gives ~3.300 and ~35.000 cells at E12.5 and E14.5, respectively. Finally, an increased deposition of ECM could also occur. However, in mature organs most of the ECM in the surrounding connective tissue is usually found at a distance from the epithelium (dense connective tissue, rich in collagen fibers) and not in close proximity (loose connective tissue, rich in cells) to the epithelium.

5. Introduction – I suggest to elaborate a bit more about tubulogenesis and lumen formation (e.g. 19914171). This is a key step preceding the tip-trunk stage.

In the revised manuscript, we included a paragraph about tubulogenesis in the developing

© 2022. Published by The Company of Biologists under the terms of the Creative Commons Attribution License (https://creativecommons.org/licenses/by/4.0/).

pancreas (see new Introduction section pages 3, 4).

6. The final paragraph about bioprinting is out of context. It is not clear at all why and how the data in this study will help bioprinting for cell therapy or disease modeling. Is there a reason to think that the exact structures identified here within fetal islets are applicable? If so, the authors should try and print such a structure from live sorted cells.

The paragraph about bioprinting has been removed from the Discussion section.

Reviewer 3

Glorieux et al. report on the establishment of 3D image acquisition and analyses of E12.5 and E14.5 whole mount mouse pancreatic tissues. More specifically, they study cellular interactions between pancreatic epithelium (as labelled with Pdx1) on the one hand and endothelium (as labelled with Erg or Cdh5) and mesenchyme (as labelled with Nkx2.5) on the other hand. They found that from E12.5 to E14.5 the interaction between endothelium and epithelium gets more intimate (moving from a 5 to a 2.5 µm distance). The authors also observe that this interaction is stronger in the pancreas (60-80%) than stomach (10-20%). In addition, mesenchymal cells also move a bit closer to the pancreatic epithelium (moving from a 7.5 to a 4.5 µm distance). The ratios of cell populations in 15 µm proximity to each other remain almost constant, but a trend towards an increase in endothelial cells and epithelial cells versus a trend towards a decrease in mesenchymal cells could be observed. The authors further observe that blood vessels close to the pancreatic epithelium increase in diameter, indicative of blood vascular perfusion. In sum, Glorieux et al. report on a technical advance in pancreatic imaging by using SPIM / LSFM that, however, has been applied to several other embryonic tissues (sometimes even using live cell imaging) – therefore, it is not well suited as a pure resources paper.
The findings reported are of descriptive nature and thus limit the enthusiasm to publish this report in Development, in particular, since no major insights into the cellular and molecular mechanisms of pancreatic development are reported. However, the technology could nicely be combined with manipulative experiments (not shown by the authors though) as to give the manuscript more value in terms of using the technology to better understand mechanisms in development.

Comments for the Author:

Major issues:

(1) The authors only report on pancreatic epithelial – mesenchymal/endothelial cell interactions. They should extend their study on beta cell – mesenchymal/endothelial cell interactions using the Ins2-Cre line that they used only once in their study. Does this interaction change between E12.5 and E14.5?

We understand the concern of this reviewer about the limited scope and novelty of our study. We thank him/her for the good suggestions on how to expand and improve our study. We followed the suggestion of the reviewer and focused on the developing endocrine islets and their interactions with the surrounding vessels and mesenchymal supporting lineages. The new experiments resulted into two new main figures (Figures 6 and 7) and 3 new Supplementary figures. The tissue interactions change indeed with time. We found an overall increase in vascular density around the endocrine islets as the pancreas develops, while the mesenchyme density decreases from E14.5 to E16.5 (see new Figure 7).

(2) The authors must extend their study to blood vessel perfusion. Using dextran or tomato lectin (or another lectin) they could directly show whether the blood vessels are perfused at E14.5 (as they suggest) and, more importantly, whether beta cell development or endocrine progenitor cell development happens close to perfused rather than non-perfused blood vessels. Ngn3-Cre mice along with Ins2-Cre mice need to be used.

The reviewer is absolutely correct. in a previous paper Shah et al. (2010) convincedly demonstrated using intracardiac injection of FITC-Tomato-Lectin that the vast majority of E10.5-E11.5 pancreatic blood vessels (Pecam+) are not perfused. They also showed that the number of pancreatic vessels that become perfused with blood slowly increases with time, with 90% of the vessels being Lectin^{+} after E15.5 (Shah et al. 2010). Specifically, during E12.5-E14.0 glucagon^{+}

© 2022. Published by The Company of Biologists under the terms of the Creative Commons Attribution License (https://creativecommons.org/licenses/by/4.0/).

cells tended to localize specifically near perfused blood vessels. We cited this paper in our manuscript, but not repeated the experiment. Nevertheless, we used our mouse Tg models to analyze the microenvironment (hence blood vessels) in the vicinity of developing islets (E14.5 and E16.5). We found that endothelial density is higher in close proximity with glucagon^{+} cells and this increases from E14.5 to E16.5. Careful tracking of glucagon^{+} cell clusters over successive z-slices also revealed that blood vessels are always present in close apposition with developing islets, as if endocrine islet budding was coordinated with the development of blood vessels. These new experiments are shown in new Figures 7 and S3.

(3) Figure 3E and 4 are probably the Figures with the newest data. Is it possible to manipulate these aspects, e.g. block blood perfusion in the developing pancreas and see what happens?

Unfortunately, blocking blood perfusion in the developing pancreas is not feasible *in vivo*. However, early pancreas development has been studied in transgenic mouse models that prevent endothelial development, hence perfusion. For example, mouse Flk1 (*a.k.a.* VEGF receptor 2) knockout embryos show defects in dorsal pancreatic bud emergence and maintenance of *Pdx1* expression (Yoshitomi and Zaret, 2004). Other groups have taken the reverse strategy and promoted vascularization using VEGFa over-expression. These studies showed an impact of expanding the vascular density on pancreatic cell differentiation (Lammert et al., 2001; Magenheim et al. 2011). Additionally, our groups and others have previously conducted studies on pancreas development in the absence of blood perfusion by culturing pancreatic explants *ex vivo* on microporous filters or on fibronectin (Pierreux et al., 2010; Petzold, Spagnoli 2012). *Ex vivo* cultures of 3D explants contain all the pancreatic cell types (epithelial, endothelial and mesenchymal) and faithfully reproduce pancreatic growth, branching morphogenesis, cell polarization and cell differentiation. All these events occur in the presence of mesenchymal and endothelial cells but in the absence of blood flow (perfusion) within the blood vessels. Altogether, these previous *in vivo* and *ex vivo* studies support a role for endothelial cells in pancreas development and differentiation, but independent from blood perfusion at least at early stages. In the present manuscript, we expanded the 3D analysis on endothelial cell spatial localization, which has not been studied before.

(4) The authors must state which novel conclusions they actually draw from their 3D images; such conclusions must be mentioned in the abstract.

We have revised the abstract to mention more clearly the conclusions of our study.

Minor issues:

(1) Figure 2C shows Ins2-Cre mice at E12.5, but no data on these mice at E14.5 are provided.

We have included images of Tg(Ins2-Cre;R26R-mTmG) at E14.5 in the revised Figure 2C.

(2) Is it possible to visualize the birth of beta cells using LSFM live cell imaging? This is probably not easy, but could give novel dynamic information about beta cell development.

We could not perform time-lapse LSFM imaging on whole-mount embryonic pancreas, because the samples are not transparent, but need to be fixed and cleared. However, we used our LSFM images to track glucagon^{+} cell clusters over successive z-slices along with the surrounding VEcad^{+} vessels. This analysis showed that blood vessels are always present in close apposition with the developing islets, suggesting that endocrine islet budding is coordinated with the development of the vessels. These new experiments are shown in the new Figures 7 and S3.

(3) Any idea why there is more epithelial-endothelial cell interaction in pancreas versus stomach? Is it because there are fewer endocrine cells in the stomach versus pancreas?

This is an interesting point for discussion. The different pattern in epithelial- endothelial cell interaction could be due to differences in the tissue- architecture between the two organs, the pancreas being a branching epithelium with a more complex folding than the stomach. Additionally, another plausible explanation could be related to the different organ function. The stomach is a glandular epithelium with mostly an exocrine function, the blood vessels present in the mucosa and sub-mucosa layers have mostly a nutritive function. In stark contrast, the pancreas is an amphicrine gland with an endocrine function and, therefore, necessitates a dense

© 2022. Published by The Company of Biologists under the terms of the Creative Commons Attribution License (https://creativecommons.org/licenses/by/4.0/).

vascular network in contact with endocrine cells for collecting hormones from the islets of Langerhans.
Alternatively, the difference in epithelial-endothelial cell interaction could be due to the different expression of the angiogenic factor, Vegfa. Indeed, starting from E14.5, Vegfa expression is higher in the trunk and branches of the pancreas as compared to the stomach epithelium (see Figure 2C,D in Pierreux et al. 2010). This angiogenic signal might promote the recruitment and expansion of an endothelial cells around the pancreatic epithelium and, consequently, induce more intimate contacts.
Finally, it is also possible that the environment in which the blood vessels develop in the pancreas and the stomach are different. For instance, the stomach epithelium might promote the development of dense connective tissue in the mucosa and submucosa, thereby maintaining blood vessels at a distance from the stomach epithelium. We have included these different possible explanations in the Discussion section of the revised manuscript.

Second decision letter

MS ID#: DEVELOP/2021/199655

MS TITLE: Development of a 3D atlas of the embryonic pancreas for topological and quantitative analysis of heterologous cell interactions

AUTHORS: Laura Glorieux, Aleksandra Sapala, David Willnow, Manon Moulis, Anna Salowka, Jean-Francois Darrigrand, Shlomit Edri, Anat Schonblum, Lina Sakhneny, Laura Schaumann, Harold F Gomez, Christine Lang, Lisa Conrad, Fabien Guillemot, Shulamit Levenberg, Limor Landsman, Dagmar Iber, Christophe E PIERREUX, and Francesca M Spagnoli
ARTICLE TYPE: Techniques and Resources Article

Dear Dr. Spagnoli

I am happy to tell you that your manuscript has been accepted for publication in Development, pending our standard ethics checks. Where referee reports on this version are available, they are appended below. The referees had some remaining minor comments that they felt would help the manuscript and it would be appreciated if you addresses this in the final version of the text.

Reviewer 1

Advance summary and potential significance to field

The authors have been responsive to the reviewers' comments and performed additional experiments and analyses. They notably added late developmental time points E16.5 and P0 and a new section on the formation of endocrine islets (line 306). This section adds to our understanding of the islet microenvironment notably the relation between islets and blood vessels. The quantitative analysis will form a solid basis for future studies. Fig. 7 also illustrates the data in an interesting way enabling a mental representation. More generally, the authors can be commended for contributing a resource that will be shared with the community. Moreover, the thorough approach, including statements on the limitations (for example methods employed to determine mesenchymal cells on line 280) cross validations between 2D and 3D (for example on line 294) is most appreciated and sets quality standard that the journal "Development" cares about.

Comments for the author

Minor criticisms:
- adding the cell numbers found at E16.5 and P0 to figure S1 would have been most useful for a complete resource.
- In figure 6 or a supplementary figure, adding higher magnifications may better illustrate the relationships between blood vessels, alpha and beta cells.

© 2022. Published by The Company of Biologists under the terms of the Creative Commons Attribution License (https://creativecommons.org/licenses/by/4.0/).

This would lend more support to the text statement: on lines 323-324 “glucagon+ alpha cells bud out as units, forming a layer of cells that remain at the periphery of the peninsula”
- It would be useful to comment on the presence of blood vessels inside the islets, even if there are none.

Reviewer 2

Advance summary and potential significance to field

The authors have largely addressed my concerns. I support publication in Development.

Comments for the author

Congratulations on a nice study and an important contribution to the community.

Reviewer 3

Advance summary and potential significance to field

The authors added two more main Figures and additional Supplemental data to strengthen their manuscript and report new findings on cell-cell interactions in 3D during pancreatic development. In particular, they added more data on the endothelial - pancreatic endocrine cell interactions, showing that interactions between endocrine cells and smaller vessels (in particular capillaries) get more and more during embryonic development. Even though the data are still descriptive, they use novel techniques and reveal cellular interactiosn in the context of pancreatic development at a very high resolution and in 3D (not described in such depth before).

Comments for the author

The authors did not add time-lapse imaging nor did they manipulate blood flow, as suggested by this referee.
Therefore, they shall instead at least mention flow-related angiocrine signalling events (see e.g., Lorenz et al., Nature 2018) as a possibility for endocrine pancreatic - vascular interactions in their Discussion, since blood flow starts at the time when islets develop and capillary-endocrine cell interactions are the strongest.

© 2022. Published by The Company of Biologists under the terms of the Creative Commons Attribution License (https://creativecommons.org/licenses/by/4.0/).
